# Detection of the Elite Structure in a Virtual Multiplex Social System by Means of a Generalised *K*-Core

**DOI:** 10.1371/journal.pone.0112606

**Published:** 2014-12-26

**Authors:** Bernat Corominas-Murtra, Benedikt Fuchs, Stefan Thurner

**Affiliations:** 1 Section for Science of Complex Systems, Medical University of Vienna, Spitalgasse 23, A-1090, Vienna, Austria; 2 Santa Fe Institute, 1399 Hyde Park Road, 87501, Santa Fe, New Mexico, United States of America; 3 IIASA, Schlossplatz 1, A-2361, Laxenburg, Austria; Centre de Physique Théorique, France

## Abstract

Elites are subgroups of individuals within a society that have the ability and means to influence, lead, govern, and shape societies. Members of elites are often well connected individuals, which enables them to impose their influence to many and to quickly gather, process, and spread information. Here we argue that elites are not only composed of highly connected individuals, but also of intermediaries connecting hubs to form a cohesive and structured elite-subgroup at the core of a social network. For this purpose we present a generalization of the 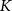-core algorithm that allows to identify a social core that is composed of well-connected hubs together with their ‘connectors’. We show the validity of the idea in the framework of a virtual world defined by a massive multiplayer online game, on which we have complete information of various social networks. Exploiting this multiplex structure, we find that the hubs of the generalised 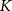-core identify those individuals that are high social performers in terms of a series of indicators that are available in the game. In addition, using a combined strategy which involves the generalised 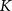-core and the recently introduced 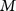-core, the elites of the different ’nations’ present in the game are perfectly identified as modules of the generalised 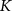-core. Interesting sudden shifts in the composition of the elite cores are observed at deep levels. We show that elite detection with the traditional 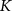-core is not possible in a reliable way. The proposed method might be useful in a series of more general applications, such as community detection.

## Introduction

**Figure 1:**
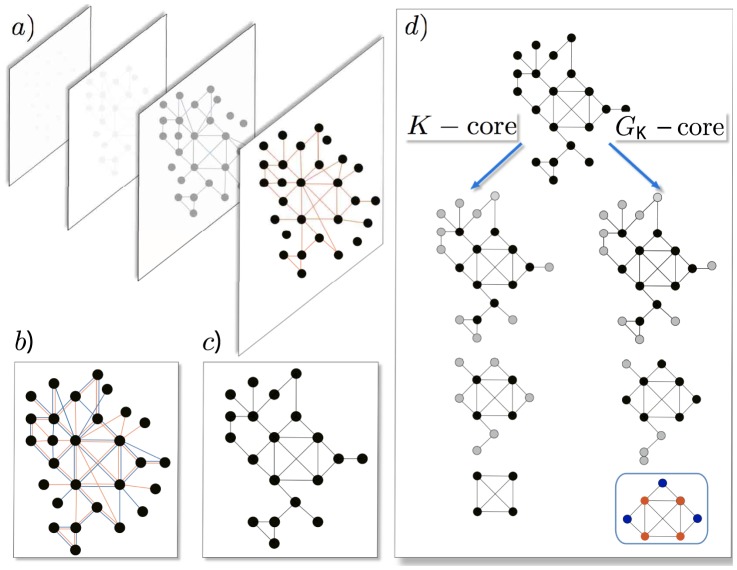
Extracting the core of a Multiplex System. (a) Representation of multiplex network (MPN) composed of several layers of different relations among nodes. (b) A MPN consisting of two link-types *orange* and *blue*, and (c) its *intersection graph* obtained by keeping those links that are present on both networks. (d) Comparison of the 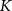-core, left and the *generalised* 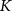-core, right algorithms, when applied to the intersection graph: while the 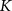-core iteratively removes those nodes whose degree is lower than 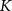, (leading to the 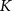-core), the 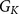-core iteratively removes nodes whose degree is lower than 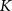 which are not connected to more than one node whose degree is equal or higher than 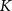. We highlight the *connectors* (blue) and the hubs (orange). Although connectors nodes may have a low degree, they play a role in keeping the overall connectivity at deep levels of network's organization.

Almost universally, across cultures and times, societies are structured in a way that a small group of individuals are in the possession of the means to influence, shape, structure, lead, and govern large proportions of entire societies. These selected minorities form the *elites*. The definition and characterization of an elite is a highly multidimensional and debated problem [1]–[5]. It incorporates considerations about wealth, experience, fame, influence over other individuals, role in societies, clubs, parties, etc. In any case elites can not be defined *per se*, but only within the context of a social system, which are superpositions of various time-varying social networks, so-called multiplex networks (MPN) [6]–[8]. These networks represent interactions between individuals as links of different types such as communication, trading, friendship, aggression, etc., see Fig. 1a. It seems natural that elites have to be defined through their location within these MPNs. Indeed, one would generally expect that members of elites are characterized by a large *connectivity* [9] in the various networks of the MPN, which enables them to exert their influence on a large number of other individuals. A large connectivity, paired with a strategic position within the MPN, also allows them to collect, process, and spread information that is of relevance to them [10]. In this view elites are ‘core-communities’ that, to a certain extent, organise the whole topology of social interactions in a social system [9]. It is further intuitive that elites are not simply a collection of highly connected individuals, but communities of individuals densely connected (a *cohesive subgroup*) containing hubs and maybe other individuals playing functional roles within such elite structure. Moreover, relations among elite members are not incidental: they are defined at the same time at multiple levels, spanning from personal and commercial relationships to information exchanges. The cohesiveness of this group can be achieved by means of direct relations among the elite members or by means of intermediaries, individuals who, although not very connected themselves, establish and coordinate the relations between well connected elite members [11]. We refer to these intermediaries as *connectors*.

Given the above considerations, the question arises if one could identify the elite members of a given society from its MPN only by topological means. The identification of cohesive subgroups at the core of social networks has a history of decades and includes the 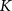-core decomposition [12]–[14], the clique identification [15], [16] or the rich club analysis [17], among other general methods of cohesive subgroup identification [18], [19]. In general, these decomposition schemes are focused on the features of the organization of hubs. However, to adequately describe the organization of a social system, one might think of alternative definitions of ‘core’, taking into account other *functional* properties of nodes than just their degree. In the spirit of our definition of elites, connectors should be included in the definition of a core. The heart of this paper is to suggest a generalization of the 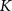-core algorithm that naturally takes the ‘functionality’ of connectors into account, and thus allows to detect cores which are composed of hubs together with their connectors. The *generalised* 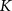-core is obtained by an iterative method inspired both by the so-called 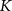-scaffold [20], [21], and the 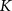-core [12], [14]. Specifically, the *generalised* 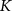-core (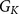-core) is the maximal induced subgraph whose nodes either have a degree larger or equal than 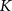 or *connect* two or more nodes with a degree larger or equal to 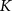, see Fig. 1b and methods for details. We will show that 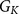-cores isolate the elite communities much more reliably than the traditional 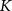-cores. Moreover, as we shall see, 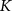-cores and 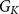-cores show substantial differences in their composition and architecture.

The quantitative exploration of structural patterns in real social systems is usually hard or even impossible due to poor data availability and due to factors that escape experimental control. Virtual societies such as those formed in Massive Multiplayer Online Games (MMOG) [22] offer an excellent opportunity to avoid these complications and allow for the first time a fully quantitative and empirical understanding of social systems under controlled conditions. Log-files of these games provide complete datasets where practically all actions and interactions of all avatars in the games are recorded. MMOGs provide a unique framework to test quantitative hypotheses and formulate entirely new questions on social systems. Data then can provide answers at unprecedented levels of precision in the social sciences. In this paper we will use data from the MMOG society of the game ‘Pardus’ (http://www.pardus.at) [23], an open-ended online game with a worldwide player base which currently contains more than 420,000 people. In this game players live in a virtual, futuristic universe where they interact with other players in a multitude of ways to achieve their self-posed goals. A number of social networks can be extracted from the Pardus game, leading to the first realization of an entire MPN of a human social system. The MPN consists of the time-varying communication, friendship, trading, enmity, attack, and revenge networks. These networks are tightly related and mutually influence each other as it has been systematically explored and quantified in [7], [23]–[28]. Here we focus on networks representing *cooperative* interactions, namely, *friendship* (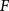), *communication* (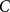) and *Trade* (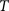). Our social system is therefore given by the MPN 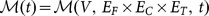, being 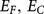 and 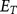 the sets of links defining a friendship relation, a communicative exchange or a commercial relation, respectively. To ensure the relevance of our results, we will filter the players to rule out the non-active ones. Specifically, we will build the nets over the most active players ‘Artemis’ universe of the game, which leads us to a set of 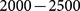 players.

It is not *a priori* clear which link type of the MPN or which combination of links is most relevant for elite detection. A communication link between two individuals might signal an occasional interaction, whereas if a communication link is paired with a trade link, this might be an indication for a much stronger relation between them. For this purpose we derive four more networks, the *intersections* among levels of the MPN, see Fig. 1a,c and methods. In these networks a link exists if it is present in two or three of the MPN layers. For these intersection graphs, we formally write 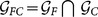, 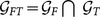, 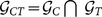 and 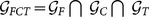. The links of these networks, often called *multi-links* [29], encode strong relationships among individuals, for they connect players interacting in more than one type of relation. The strongest links in this sense are those in 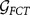, a graph which we refer to as the structural *backbone* of the multiplex system. The identification of elite structures and core organization is based on the 3 networks of the MPN and their associated four intersection graphs.

The core organization of 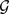 will be explored explicitly by computing the sequence of 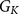-cores, the so-called 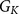-decomposition sequence, which amounts to a ‘russian doll’ decomposition of the networks, 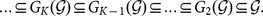
The behavior of this sequence of nested levels of networks (either seen in terms of the statistical properties of their graphs, or from their social composition) is essential to identify the elite organization and the elite structure of our virtual social system. When compared to the traditional 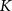-core, we will see that the 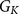-core provides a much more detailed picture of the nested community structures. Data from the ‘Pardus’ game enables us to test and compare the quality of the identified core and to see to what extend it relates to properties that are expected for an elite. For every player we have a record of wealth, leadership role in local organizational structures, and importance in leadership as measured by a ‘global leadership index’. Local organizational structures are clubs, societies and political parties, in which players organise; we know which player has a leading role in that local organization which can be president, treasurer or application master. The global leadership index is a status index that is assigned to each player (visible to all the others) which increases when special tasks (missions) are fulfilled. Such an index is an indicator of the potential influence of the player on decisions affecting the whole ‘faction’ it belongs to. A faction would correspond to a country in the real world. In its current state, the game extends over a universe containing three factions, which are politically independent and lead by their respective elites.

A final word of caution is needed, in relation to the significance of the data shown here. Since there is no formal/topological definition of elite in a given multiplex society, we adopted the position of showing the averages of the indicators of social relevance of the different core subgraphs we isolate. We checked the position of the topologically isolated sets of nodes within the raw rank of social performance of all players under study. However, an elite is not just a *list* of the best performers but a cohesive social structure. Therefore, rigorous indicators of statistical relevance would imply the assumption of meaningful null models. This is undoubtedly extremely interesting, but it is an issue going far beyond the scope of this paper. Instead, we adopted the position of giving relevance to our results by confronting them the the ones obtained by means of the K-core, the standard core extraction mechanism, originally designed to extract the network substructure of the most influential individuals in a given society.

## Results

We extract the mentioned seven networks from the Pardus data, in the same way as described in [7], [23]. Our analysis is performed over the three networks 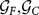 and 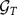 obtained from the most active players in two time spans of sixty days, 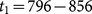 and 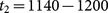 in units of days since beginning of the game. A link between two players in the layer 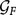 exists if at least one player recognises the other as ’friend’ in the whole studied period. Likewise, a link between two players in the layer 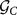 exists if at least one player has sent a message to the other in the studied time span. Finally, a link between two players in 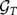 exists if there has been at least one commercial transaction between these two players within the studied time span. The set of players that will define the set 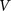 of the MPN obtained from the period 796–856 contains 2422 players, whereas the set of players defining the MPN of the period 1140–1200 comprises 2059 players. Chosen players are those who are active in at least all three levels of the MPN during all the studied periods. The periods have been chosen using two criteria i) The periods are chosen far away enough from the starting of the game, to ensure that the social structure of the virtual society achieved certain degree of maturity and ii) The comprised time spans do not contain ‘war’ periods, which may introduce an extra source of noise.

The results of the two time periods under study show a remarkably similar behaviour. Therefore, throughout this section we will mainly show the numerical values of the time period 1140–1200, for the sake of readability. In the supplementary material the reader can find a systematic analysis of the two periods under study.

### The backbone exhibits high levels of clustering

The statistical analysis of networks shows remarkable degree of clustering at all levels of description. In the period 1140–1200, the average degrees for the various layers of the MPN are 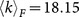, 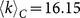, and 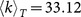 and the clustering coefficients are remarkably high if we take into account these connectivities: 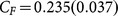, 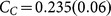, and 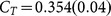. Numbers in brackets correspond to the expected value of the clustering coefficient in an ensemble of random networks having the same size and degree distribution than the real ones, see methods and S1 File. The intersection networks show a slight decrease on the number of nodes (see Table S1,S2 in S1 File) and smaller average degrees: 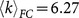, 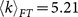, 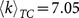, and most pronounced, 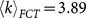, as expected. Although the average degree is lower than in the MPNs, the clustering coefficients still show remarkably high values, especially when compared with the randomized values, 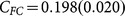, 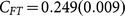, 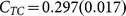, and 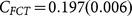. The persistence of the clustering coefficient, even for 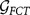, where the expected 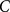 for the randomized case almost vanishes, indicates that the mechanism of *triadic closure* [30]–[33] plays an important role in the dynamical formation of the backbone structure in social systems.

### The 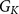-sequence

We compute the 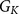-decomposition sequence (see S1 File for details) and observe the following trends. We generally observe long 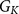
- decomposition sequences. The length of the decomposition sequence is the largest value of 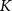 for which 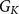-core is not empty. For the different networks 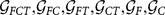 and 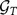, these limit values are found at 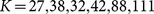 and, again 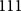, respectively.

**Figure 2:**
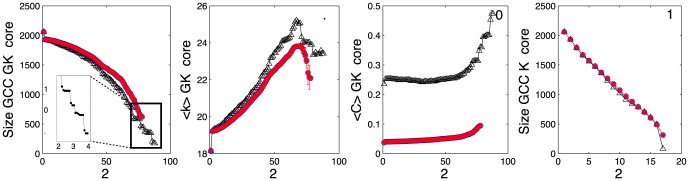
Evolution of the topological indicators along the 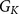-decomposition sequence for the 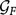 level of the MPN of the period 1140–1200. In a) we have the evolution of the size of the 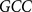 of the 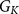-core of the net (black) and its randomized counterpart (red). In the box inside the figure we highlight the evolution of the size of the 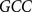 of the 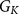-core at high 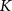-levels, where flats regions followed by sudden decreases are observed. b) Evolution of the average degree of the 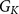-core (black) and its randomized counterpart (red). c) Evolution of the average clustering coefficient of the net (black) against its randomized counterpart (red). Finally, in d) We plot the evolution of the 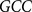 of the 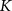-core of the net in terms of 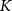 (black) against its randomized counterpart (red). Observe that, for this latter plot, there are no significant statistical differences on the behaviour of the real graph when compared to the randomized one. The results for the random counterpart of the net have been obtained from an ensemble of 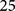 randomized versions of 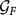, see text and methods section.

In Fig. 2a the size of the *giant connected component* (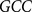) [34] along the 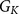-decomposition sequence is shown for the 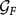 network (black) -In a little abuse of notation, we refer to the 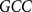 as the set of nodes that from a connected component significantly larger than the others, if there exist any. In our case, the 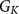-cores generally show a single connected component. We observe that the 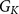-decomposition sequence is longer than the one expected by chance, see Fig. 2a, (red). The situation for the traditional 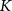-core is different, with a behaviour similar to the one expected by chance in all studied subgraphs, see Fig. 2d. Further, the evolution of the size 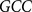 of the 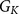-cores shows plateaus followed by abrupt changes, which may depict different levels of core organization. On closer inspection, we find that often these changes signal the collapse of a cluster, which forms a cohesive community at certain level 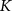, and which is completely absent at level 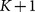. The structure of the 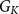-core just before a collapse represents one organizational level which is replaced by a deeper one, maybe with different topological and social characteristics. We observe that the length of the decomposition sequence strongly depends on the size of the network, a feature probably due to the power law degree distribution they exhibit. As shown in [21] for generic sequences of nested subgraphs, the depth of the decomposition sequence diverges for this kind of networks.

The evolution of the average degree 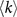 along the decomposition sequence for the 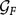 network is seen in Fig. 2b (black). We find significant differences between the social networks and their randomized counterparts (red). In most cases one observes that the average degrees along the decomposition sequence first increase with 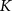, revealing a phenomenon which resembles the so-called *rich club* [17]. Here, elements of the 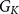-core tend to be more connected among themselves than would be expected by chance. We find an exception in the 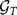 network where there are no significant differences between the real average degrees and those obtained after randomization. This increasing trend usually peaks and stops at deep levels, followed by a slight decrease at the deepest levels, see Fig. 2b. The increase is absent in standard models of random graph like the Erdös Rény [34] and Barabási-Albert [35] networks, see Fig. S1 of the S1 File. This means that the particular structure of the social network determines the functional form of this curve. Since the randomized ensembles also show an increasing trend of connectivity through the sequences, see Fig. 2b (red), one might expect that the degree distribution is partially responsible of the observed increase. Furthermore, the presence of high clustering could also be responsible for an additional increase of the connectivity of the cores, thus explaining the deviation from their randomized counterparts.

Finally, the evolution of the clustering coefficient displays two clearly differentiated regions: At low and medium stages of the decomposition sequence it shows a more or less constant behaviour, followed by an increase at later stages of the sequence. This latter increase may also be the footprint of a rich-club phenomenon in the networks under study. It is worth to observe that along the decomposition sequence, the real values of the clustering coefficient are at least one order of magnitude higher than the expected by chance. In Fig. 2c we display the evolution of the clustering coefficient along the decomposition sequence for the 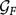 network.

### Identification of characteristic 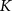-levels and core communities through the 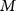-core

**Figure 3:**
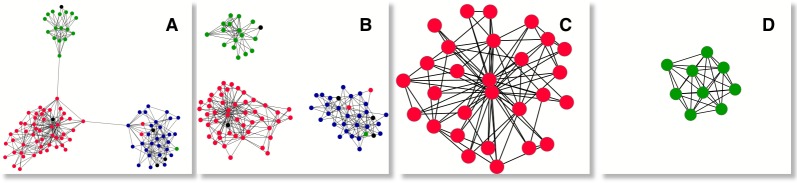
National elites define topological communities at deep levels. The composition of the 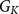-core in terms of nations reveals that the multiplex system is organised around the elites of the three existing nations, whose members are depicted with different colours (see text for the use of colours). We have a) the *characteristic* 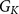 for 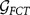, where we find that the 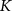-critical level is located at 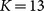 b) after the application of the 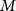-core (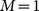), three components appear isolated, to be identified as the three communities composing the 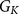-core. Such communities are almost uniformly populated by members of the same nation. In c) we have the deepest 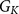-core, which contains members of only one nation. Interestingly, the composition of the deepest 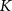-core of the 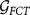, 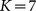, d), is absolutely different from the composition of the deepest 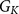-core of the same net, located at 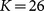, showing interesting qualitative differences between these two approaches of core extraction. All pictures belong to the period 1140–1200.

In the previous section we pointed out that the evolution of the size of the 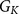-core throughout the decomposition sequence eventually displays sudden decreases, and that such sharp decays might be related to massive collapses of communities the core. Such change might reveal different levels of core organization. How to identify such crucial levels and, therefore, communities inside the 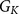-core? We assume that the cohesiveness of such communities leads to a high degree of transitivity between them, i.e., that the clustering coefficient inside such communities is exceptionally high. This intuition is supported by the extremely high clustering coefficient values found in the system under study, as we reported above. Moreover, we assume that the degree of transitivity between communities is very low namely, that connections between members of different communities are performed by simple links or by means of connector nodes. Under such defining assumptions of core community, the recently introduced 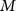-core [36] plays a crucial role. The 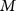-core is the *maximally induced subgraph in which each link participates at least in* 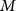 *triangles*. Therefore, the application of the 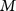-core with 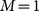, 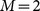 over the 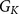-cores will remove those links (and maybe some nodes) which do no participate in a highly clustered structure, eventually acting as bridges between communities. The *unconnected components that may emerge from the application of the* 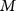 *-core (* 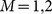 *) to the* 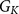 *-core will be the core communities of our graph at level* 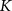, see Fig. 3a,b, methods section and S1 File for a detailed information. For the sake of readability, let us refer to the 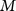-core of the 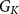-core as 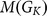. As long as 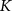 increases, the number of components of 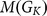 (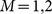) may fluctuate, thereby identifying different organizational levels within the core of the network. Such fluctuations, if any, will define different levels of core organization. In general, the deepest cores of the networks under study display only a single component, and we will put our focus on the last 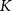 by which 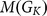 (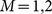) contains more than a single component. We will refer to this level of organization as the *characteristic* 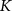 *-level of organization*. It may happen that such a level does not exist, then we will conclude that for this network and under our assumptions, the 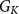-core does not change dramatically its structure throughout the values of 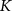. The rationale behind the definition of this characteristic level is clear: we want to study the structure of the core before the last reorganization, for it may contain many topological and properties absent in the deepest one. As we shall see, this methodology is able to perfectly identify core communities in our system, see Fig. 3a,b. It is worth to emphasise that randomized versions of the nets under study always display a single component and no communities –and, thus, no characteristic 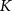-levels– can be identified.

With the *characteristic* 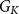-core and the *deepest* 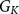-core, we have two snapshots of the core organization, presumably depicting different structural features. The former represents a core structure which vanishes at deeper levels, the latter shows how the elements at the deepest level of description are organised. For the networks corresponding to the period 1140-1200, 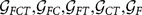, we got the following characteristic 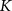-levels: 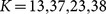 and 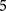 respectively. 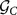 and 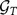 did not show any characteristic level. The networks obtained out of the intersection of MPN levels display a clearer core community structure and thus relevant characteristic levels can be identified. In the case of 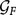, the characteristic level is found at a very low 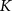, so its statistical relevance is lower than the characteristic 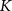-levels reported for the intersection nets.

### The 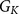-core and the elites of the social system

We can now characterize the individuals populating the cores of the various networks with a series of quantitative social indicators in the ‘Pardus’ society. These measure status, competence, social leadership, relevance and success of various kinds. In particular we use the following indicators, and we indicate how they appear in Table 1: *Experience* (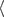 Exp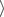, in the table. Numerical indicator accounting for the experience of the player), *Activity* (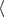
Act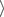 in the table. Number of actions performed by the player), *Age* (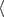
Age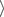 in the table. Age in units of days after the player joined the game), *Wealth*, (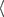
Wealth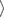 numerical indicator accounting for the wealth of the player within the game), *Fraction of leaders* (FracL, in the table. Fraction of players who are leaders in some aspect in a given subgroup of the society at the local level), and *Global leadership* (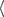
GlobL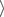 in the table. Numerical indicator evaluating the degree of leadership of the player). For detailed information about the definition of these indicators, see S1 File. We finally checked the *gender composition*, the fraction of male/female players in the core. We classify the nodes in the core whether they are a hub or a connector, and present results accordingly. We also computed the scores obtained by the members belonging to the deepest 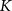-core, of each studied graph. In Table 1 we show the scores from four networks 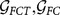, 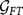 and 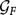, see S1 File for Tables with all social indicators over core subgraphs obtained from all networks belonging to the two periods under study.

**Table 1:** Social indicators of the isolated groups of nodes.

	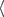 Exp 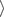	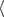 Act 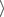	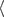 Age 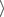	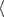 Wealth 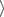	gComp	FracL	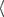 GlobL 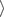	N
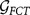								
Char. 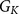	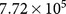	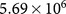	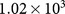	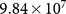	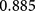	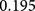	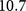	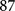
Hubs	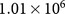	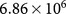	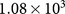	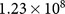	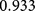	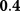	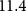	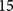
Deep. 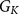	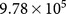	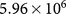	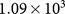	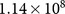	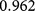	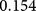	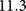	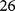
Hubs	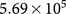	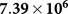	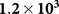	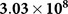	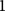	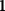	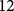	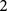
Deep. 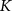 -Core	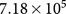	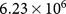	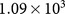	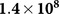	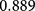	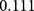	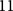	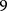
All Net	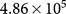	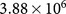	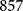	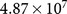	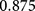	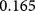	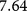	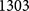
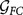								
Char. 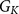	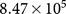	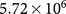	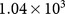	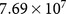	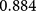	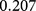	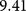	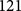
Hubs	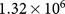	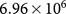	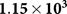	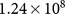	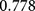	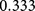	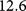	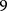
Deep. 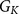	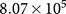	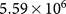	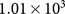	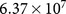	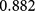	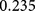	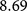	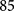
Hubs	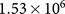	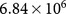	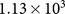	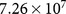	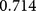	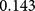	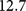	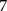
Deep. 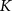 -Core	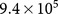	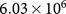	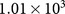	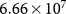	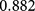	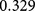	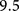	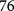
All Net	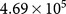	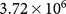	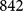	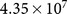	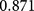	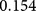	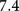	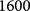
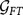								
Char. 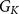	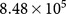	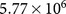	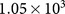	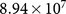	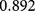	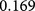	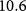	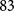
Hubs	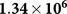	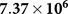	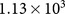	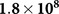	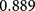	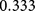	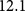	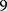
Deep. 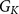	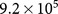	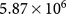	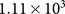	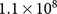	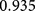	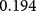	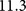	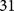
Hubs	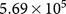	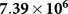	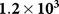	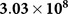	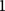	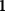	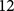	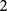
Deep. 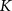 -Core	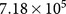	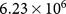	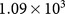	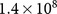	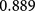	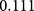	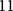	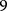
All Net	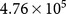	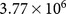	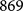	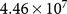	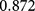	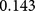	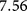	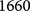
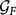								
Char. 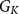	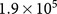	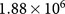	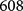	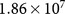	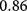	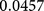	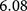	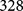
Hubs	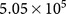	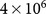	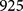	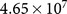	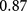	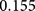	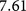	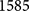
Deep. 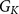	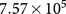	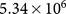	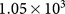	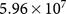	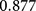	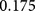	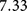	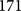
Hubs	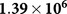	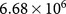	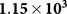	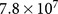	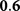	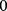	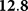	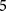
Deep. 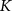 -Core	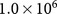	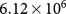	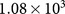	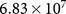	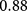	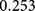	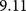	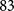
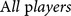	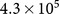	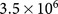	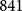	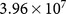	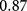	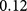	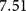	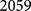

We show the scores for the cores of the 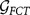, 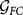, 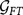
and 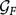 networks. ‘Char. 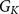
’ refers to the connectors of the *Characteristic* 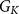, ‘Hubs’ below it refers to *Hubs of the Characteristic* 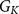. ‘Deep. 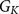
’ refers to the connectors of the *Deepest* 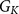. ‘Hubs’ below it refers to *Hubs of the Deepest* 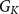. Deep. 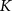-core refers to the nodes of the *Deepest* 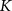 *-core*. ‘All net’ refers to all players belonging to the net whose results for the different cores is shown immediately above.We highlighted in boldface the two highest average score for each indicator.

**Figure 4:**
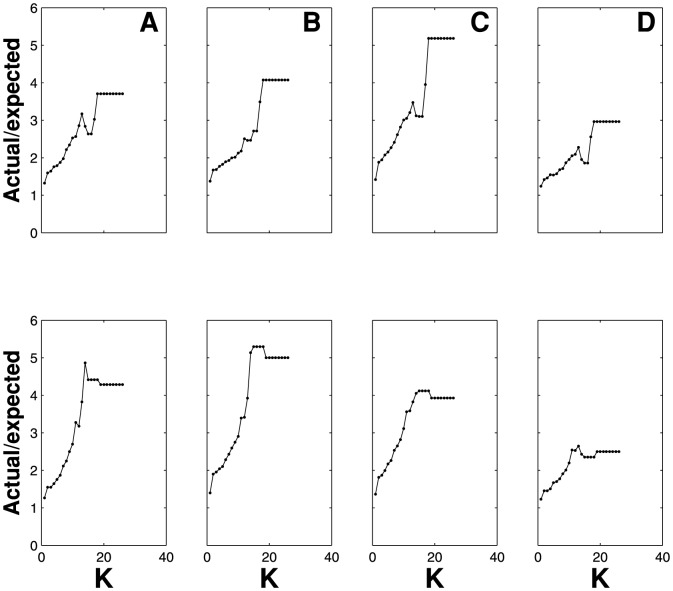
Overabundance of members of the 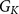-core in the set of the top-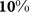 best performers of the game. In these plots we show the evolution along the 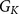-decomposition sequence of the quotient between the actual number of members belonging to the 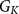-core which also belong to the set of the top-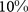 best performers of a given indicator against the expected number of them in case they are spread randomly. On top we have the results for the period 756–856 and at the bottom we have the results for the period 1140–1200, both for the 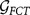 networks of their respective periods. We plot this ratio for a) Wealth, b) Global leadership, c) Activity and d) Experience. All of them show an overabundance of members of the 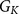-core, showing an intrinsic relation between better social performance and deep 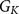-core membership. It is worth to observe i) the clear overabundance of members of the 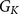-core within the set of the top 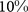 in any indicator and ii) the change of the trend after the characteristic 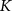-level, which is 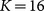 for the 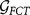 of the period 796–856 and 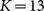 for the period 1140–1200.

The combination of the filtering provided by the intersection plus the 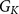-core extraction clearly identifies the structured groups of players having the highest indicators of social performance and influence. Although, as we pointed out above, there is no null model for an elite detection, one can analyse how relevant are the nodes of the topologically isolated graphs within the collection of raw values of performance indicators belonging to all players of our MPN. Indeed, let us rank all players of the MPN with respect to their performance in a given indicator and then take the 10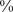 best performers of such indicator. Then, to check if the nodes of our subgraphs are among the best performers we compare the actual number of members which belong both to a given 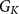-core and to this top-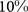 set of players against the expected number of players belonging to the 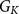-core who also belong to this top-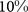 set. What we observe is that, both for wealth and global leadership, the actual number of players of a given 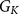-core which belong to the set of top-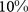 best performers scales up to 5 times the expected one, which shows that there is a strong relation between good performance within the society and being member of the 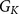-core. In Fig. 4 we show the ratio between the actual number of members of the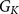-core belonging to the top-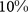 against the expected value. We show the evolution of such ratio for the two periods under study for *global leadership*, Fig. 4a, *Wealth*, Fig. 4b, *Activity*, Fig. 4c, and *Experience*, Fig. 4d. All plots show an increasing trend which stops around the characteristic 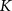-level. Beyond this, the trend flattens and becomes stable, due to the very tiny variations suffered by the 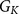-core at these levels, until it completely collapses.

In table 1 we highlighted in Boldface the two highest average scores for the following sets of nodes: Connectors of the 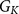-core at the characteristic 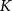-level, Hubs of the 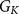-core at the characteristic level, Connectors of the deepest 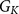-core, Hubs of the deepest 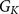-core and the scores of the players of the whole network. We show the results for 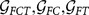 and 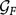 for the period 1140-1200. In tables S1 and S2 of the S1 File the reader will find an exhaustive analysis of all the nets belonging to the two periods under study. Interestingly, the highest scores of a given network are not necessarily found at the deepest level of the decomposition sequence, but are usually found in the identified characteristic 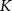-level, as seen in Table 1 in *Experience* in 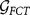 and *Wealth* in 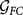. This happens even though the number of players belonging to the characteristic 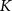-level is substantially larger than the number of players populating the deepest 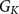-core.

We finally check if the membership to the connector set of a 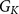-core implies a distinction with respect to those players whose connectivity patterns are comparable. Specifically, we refer to individuals having the same degree than a given connector but not being members to the connector set of 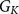. Suppose that an individual 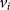 is a connector in the characteristic 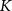-level of 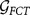, (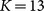, for the period 1140-1200) with a degree in the 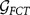 network of 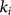. Now take all individuals in 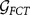 whose degree is equal to 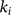 but who *do not* belong to the characteristic 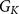 of this net. We observe that the relative performance of connectors with respect to those associated non-connectors of same degree is about 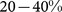 higher, in particular: 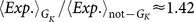, 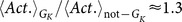, 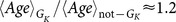 and 
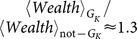
. These results point to the fact that to belong to the 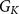-core structure increases the chances of having high scores of social performance. In some cases, we observe that the performance of connectors of the deepest 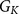-core is still higher than the one exhibited by the members of the 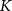-core, see, for example, 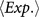 for 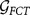 in Table 1 and S1 File. Therefore, connectors, although in general they perform worse than hubs in the 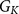-cores, could constitute a secondary elite, which presumably takes advantage of the knowledge of the underlying net of relations defining the dynamics of the social system.

### 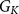-core clusters identify national elites/sharp reorganization at deep levels

**Figure 5:**
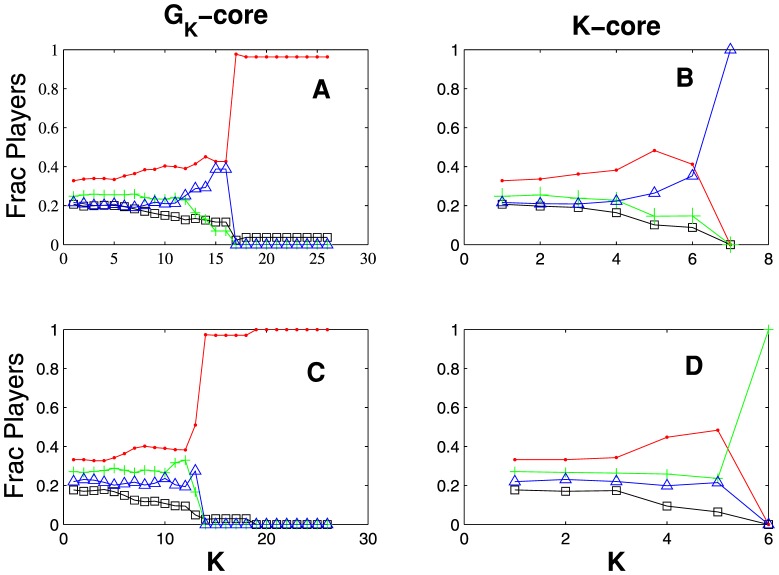
Sharp transitions at the core organization of social networks. The value at 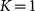 belongs to the composition of the society at the time period under study. On top a) we have the nation composition of the 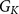-core and c) the 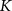-core as a function of 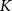 for the 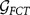 network corresponding to the period 796–856. At the bottom b) we have the nation composition of the 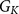-core and (d) the 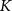-core in terms of 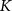 for the 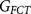 network corresponding to the period 1140-1200. Colours depict the different nations. As long as 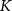 increases, the composition of the cores in terms of nationalities is more or less stationary, with values close to the ones we find in the whole system. At certain 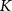 -right after the characteristic 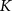
- an abrupt change is observed a) for the and 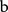), and the composition of the cores becomes uniformly populated by only one nation. The same phenomenon is observed when looking at the 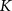-core decomposition sequence, although less pronounced. Notice that the deep 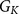-cores isolated the same nation cluster in both periods (the ‘red’ nation), whereas the 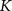-cores didn't.

We finally look at the national composition of the cores. Players usually belong to one of three ‘factions’ existing in the game, which are the equivalent of countries or nations. These nations are labeled as ‘nation 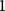
’, ‘nation 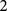
’ and ‘nation 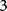
’, associated to colours red, green and blue, respectively, in Figs. 3 and 5. Players shown in black are not associated to any nation. Over all the population of the *Artemis* universe, the fraction of players in each nation is 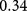, 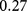 and 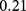, for nations 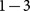, respectively. Players not associated to any nation represent a fraction of 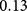 of all players.

Along the 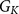-decomposition sequence of all studied networks, the nation composition of the 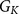-cores displays two well differentiated regions. At lower levels of 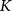, the national composition of the 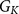-core is close to the one corresponding to the whole society. At high 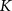-levels, 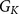-cores are populated only by members of a single nation. The shift between these two qualitatively different core organizations is abrupt, and occurs right after the characteristic 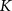-level. This behavior can be clearly seen in Fig. 5a,c, where we plot the evolution of the national composition of 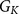-cores along the 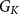-decomposition sequence of 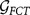 belonging to the two periods under study. The evolution of the national composition of the 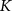-core also show a similar behaviour, although less abrupt and only at the very late stages of the 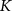-core-decomposition sequence, see Fig. 5b,d.

The application of the 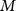-core (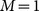) over the 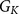-core shows that the elites of the three nations are clearly identified as clusters at the characteristic 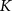-level. This can be seen in Fig. 3a,b, where we have the 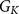-core 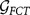 at the characteristic 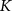-level and the 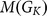. As we can see, the proposed method combining the 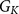-core and the 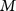-core perfectly identifies three communities belonging to the three existing nations. Interestingly, the cohesion of the entire core structure across nations is assured only by connectors. At deeper 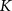-levels, only members of one nation populate the 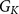-core, forming a compact cluster with no community differentiation, see Fig. 3c. The deepest 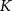-level of the 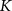-core is also populated by individuals belonging all of them to the same nation, see Fig. 3d. It is worth to remark that, against intuition, the national cluster isolated by the deepest 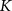-core differs completely from the one isolated by the deepest 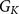-core. Finally, it is worth to mention that 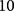 of the 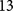 identified hubs of the characteristic 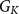-core of 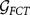 have a specific leadership role, whereas only 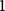 of the 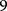 members of the deepest 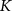-core does.

## Discussion

The aim of this study was to propose a topological method to detect the elites in a social system. We define elites not only as the set of highly connected individuals within a society, but as the set of highly connected ones *together* with their connectors in a network whose links depict multiple relations, like personal, communication or trade ones. Those elites are, presumably, strategically located at the core of the multiplex system defined by the society. To identify the elite cores, we suggest an algorithm that is similar in spirit to the traditional 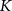-core, but that leads to entirely different compositions of the resulting core, which we called the *generalised* 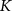-core. As a test system we used the human society of players of the MMOG Pardus, which not only provides the networks of various social interactions [7], [23]–[27], but also contains quantitative information of how individual players perform socially within the society in terms of leadership, wealth, social status among other skills, in which elite members are expected to score exceptionally high. We find that elite structures are formed by hubs connected either directly or through connectors, generally at deep levels of the core (large 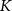). Hubs of these core subsystems display the highest scores on social relevance, and this is especially true for the backbone network and for the networks obtained out of the intersection of two levels of the MPN, specifically, of friendship and communication levels, and of friendship and trade levels. In addition, we could show that connectors within the 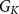-core perform consistently worse than hubs, however, we collected evidence pointing to the fact that connectors clearly socially outperform individuals (matched for their degree) that are not part of the 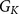-core. This indicates that connectors could constitute something like a ‘secondary’ elite within the system, taking advantage of the knowledge they have of the underlying network of social relationships. In terms of national composition and core community structure, we have seen that a combined strategy including the use of the recently introduced 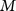-core and the 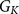-core clearly detects the clusters belonging to the elites of the three nations present in the game, thereby providing a new tool for community detection focused on the core properties of the net. Reorganization of the national composition of the cores happens in sharp bursts, rapid changes which are the footprint of the collapse of clusters within the core from one level 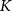 to another. In all performed analysis, it is worth mentioning the low performance of the 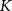-core, when compared to the 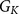-core to identify those leading subsets of individuals. We finally point out that, in spite of their low average degree, in all of the studied networks we found a remarkable level of clustering, which we attribute to the process of triadic-closure that seems to be a major driving force in the dynamics of social network formation [7], [30]–[33].

The presented results suggest that the subgraphs isolated by means of the 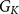-core actually correspond to the way elites interact and define cohesive subgroups. In more general terms, further works could explore the role of connector nodes in terms of information flow within networks or their presumably relevant role when a dynamical process is defined over the network. It is reasonable to think that the combination of both low connectivity and their role of hinge between clusters may provide them a predominant role in terms of dynamic organization within the network. The proposed method could lead to a wide range of more general applications, such as network visualization or as a community detection algorithm.

## Materials and Methods

### Randomisation of Networks

Random ensembles of a given network 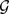 have been obtained after a rewiring process which keeps the degree of each node invariant. For a real network 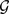, we created 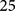 randomized versions by applying the rewiring operation 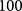 times the number of links of 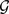.

### Intersection of different levels of the multiplex system

We formally refer to multiplex networks (MPNs) as 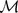, and to single graphs as 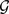. In a multiplex graph, 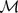, the set of nodes 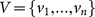 can be connected by different types of relations or links 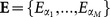, 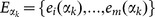. The whole multiplex is thus described by 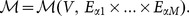
Let 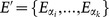, 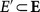, be a subset of the overall type of potential relations that can exist between two nodes, thereby redefining the concept of *link* as a collection of relations that relate two given nodes, instead of a single type of relation. We define the 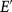-intersection network, 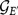 as 
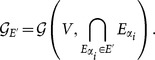
In this network, links connect those pairs of nodes which are connected through, at least, links of type 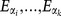.

### The generalised 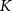-core

The *generalised* 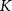-core subgraph, 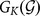 of a given graph 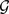 is the maximal induced subgraph in which every node is either a hub with a degree equal or higher than 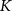, or a connector that – regardless of its degree – connects at least 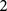 hubs with degree equal or higher than 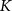. It can be obtained through a recursive pruning process. Starting with graph 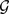 we remove all nodes 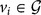 satisfying that: (1) its degree is lower than 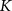 *and* (2) at most one of its nearest neighbors has a degree equal or higher than 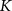. We iteratively apply this operation over a finite graph 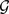 until no nodes can be pruned, either because the 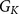-core is empty or because all nodes which survived the iterative pruning mechanism cannot be removed following the above instructions. The graph obtained after this process is the *generalised* 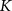-core subgraph. Note that, for any finite graph, there exists a 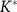 by which even though 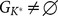, 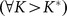 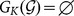. We refer to 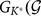) as the *deepest* 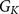-core of the network 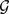, see S1 File for the algorithm.

The standard 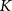-core is obtained by means of an iterative algorithm like the one shown above. The step of the algorithm consists in removing nodes whose degree is lower than 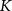. This is performed iteratively until there are no more nodes to prune, see S1 File.

Finally, the 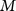-core is obtained by means of an iterative algorithm like the ones shown above. The step of the algorithm consists in removing *links* participating in less than 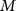 triangles. Again, this is performed iteratively until there are no more nodes to prune, see S1 File.

### Identifying levels of organization at the core

The definition of level of organization is based on the presence of highly clustered communities in the 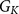-core and its eventual collapse when 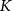 increases. Specifically, given a graph 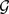:

- Compute its 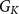-core
- Compute its 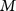-core with 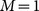 over the 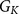-core and check if the subgraph contains more than a single component. If not, compute the 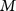-core (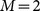) over the 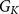-core and check if it contains more than a single component.
- Components of the 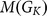 are the core communities at level 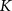 of the 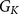-core.
- If the 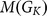 with 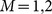 contains a different number of components than 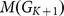 (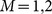), 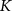 is a characteristic level of organization.

Throughout the paper we have been focused on the characteristic level of organization defined by the *largest* 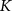 *by which* 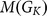, (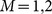) *contains more than single component.* At deep levels, all the studied 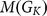
's contain only a single component. Furthermore, it may happen that 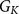 itself contains more than a single component. This does not change the algorithm for characteristic 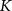-level identification.

## Supporting Information

S1 File **Rigorous definition of the algorithms.** Study of the behaviour of standard models of networks. Systematic analysis of the topological properties studied in the main text through all networks under study. Table S1 and Table S2 of average social indicators for all the studied subgraphs for both periods under study.
(PDF)

S1 Data **Data used to generate the results of the paper.** Includes: A table of indicators of social performance for each player in each period under study and the three networks of the multiplex system for each period under study.
(ZIP)
