# Supplementary material for: Detection of the Elite Structure in a Virtual Multiplex Social System by Means of a Generalised K-Core
Source: PLoS One. 2014 Dec 26;9(12):e112606. doi: 10.1371/journal.pone.0112606 (PMC4277282; doi:10.1371/journal.pone.0112606)
Supplement: S1 File — Rigorous definition of the algorithms. Study of the behaviour of standard models of networks. Systematic analysis of the topological properties studied in the main text through all networks under study. Table S1 and Table S2 of average social indicators for all the studied subgraphs for both periods under study. (PDF) [file pone.0112606.s001.pdf]

Elite structure detection in a virtual multiplex social system by means of a generalized K-core: Supplementary Information

Bernat Corominas-Murtra[1], Benedikt Fuchs[1] and Stefan Thurner[1,2,3]
[1] *Section for Science of Complex Systems; Medical University of Vienna, Spitalgasse 23; A-1090, Austria*
[2]*Santa Fe Institute, 1399 Hyde Park Road, New Mexico 87501, USA*
[3] *IIASA, Schlossplatz 1, A-2361 Laxenburg; Austria*

I. EXTRACTING THE CORE

A. Intersection of different levels of the multiplex system

Let us have a graph $\mathcal{G}(V, E)$ where $V = \{v_1, ..., v_n\}$ is the set of *nodes* and $E = \{e_i, ..., e_m\}$ the set of *links* connecting this nodes. Given a node v_i its *degree* is the number of first neighbors, or nodes a given node is linked to, to be written as $k(v_i)$. The probability that a randomly choosen has degree k is $p(k)$. The first moment of the degree distribution gives us the *average degree* $\langle k \rangle = \sum_k kp(k)$ [1]. Social systems are better described by means of *multiplex graphs*, [2] which can be thought of as different graphs sharing the same set of nodes. In a multiplex graph, $\mathcal{M}$, the set of nodes $V = \{v_1, ..., v_n\}$ can be connected by different types of relations or links $\mathbf{E} = \{E_{\alpha_1}, ..., E_{\alpha_M}\}$, $E_{\alpha_k} = \{e_i(\alpha_k), ..., e_m(\alpha_k)\}$. The whole multiplex system is thus described by:

$$\mathcal{M} = \mathcal{M}(V, E_{\alpha_1} \times ... \times E_{\alpha_M}). \tag{1}$$

In these networks, concepts such as degree distribution or average degree are relative to the type of relations (links) we are interested in. Now let $E' = \{E_{\alpha_i}, ..., E_{\alpha_k}\}$, $E' \subset \mathbf{E}$, be a subset of the overall type of potential relations that can exist between two nodes. We define the E'-intersection network, $\mathcal{G}_{E'}$ as follows:

$$\mathcal{G}_{E'} = \mathcal{G}\left(V, \bigcap_{E_{\alpha_i} \in E'} E_{\alpha_i}\right) \tag{2}$$

In this network, links connect those pairs of nodes which are connected, at least, by links of type $E_{\alpha_i}, ..., E_{\alpha_k}$. Links in $\mathcal{G}_{E'}$ are called *multilinks*.

B. The backbone of the multiplex system

A special and particularly interesting case of equation (2) is the graph of the intersection of all types of relations, $\mathcal{G}_I$ (to be named $\mathcal{G}_{FCT}$, in the main text), which depicts the *backbone* of the multiplex system depicted by $\mathcal{M}$, namely:

$$\mathcal{G}_I = \mathcal{G}\left(V, \bigcap_{1 \le i \le M} E_{\alpha_i}\right). \tag{3}$$

We point out that we have to be careful when choosing the different sets of links $E_{\alpha_1}, ..., E_{\alpha_M}$, since antagonistic relationships (such as enmity and friendship) can lead to empty intersections. We thereby restrict the definition of the intersection graph when this is performed over *compatible* sets of links.

C. The Generalized K-core subgraph

The *Generalized K-core* subgraph of a given graph $\mathcal{G}$, $G_K(\mathcal{G})$ or G_K-core of $\mathcal{G}$, is the maximal induced subgraph within which every node is either a hub (its degree is equal or higher than K) or a connector (its degree is lower than K but it connects at least 2 hubs). Increasing the threshold K we obtain the *decomposition sequence* of $\mathcal{G}$ in terms of G_K, namely:

$$... \subseteq G_K(\mathcal{G}) \subseteq G_{K-1}(\mathcal{G}) \subseteq ... \subseteq G_2(\mathcal{G}) \subseteq \mathcal{G}.$$

We will refer to the above sequence as the *G_K-decomposition sequence* of $\mathcal{G}$. The G_K-core of a given graph $\mathcal{G}$ can be obtained through an iterative pruning process: Suppose an operation $H_K(\mathcal{G})$ by which we prune all the nodes $v_i \in \mathcal{G}$ satisfying both that

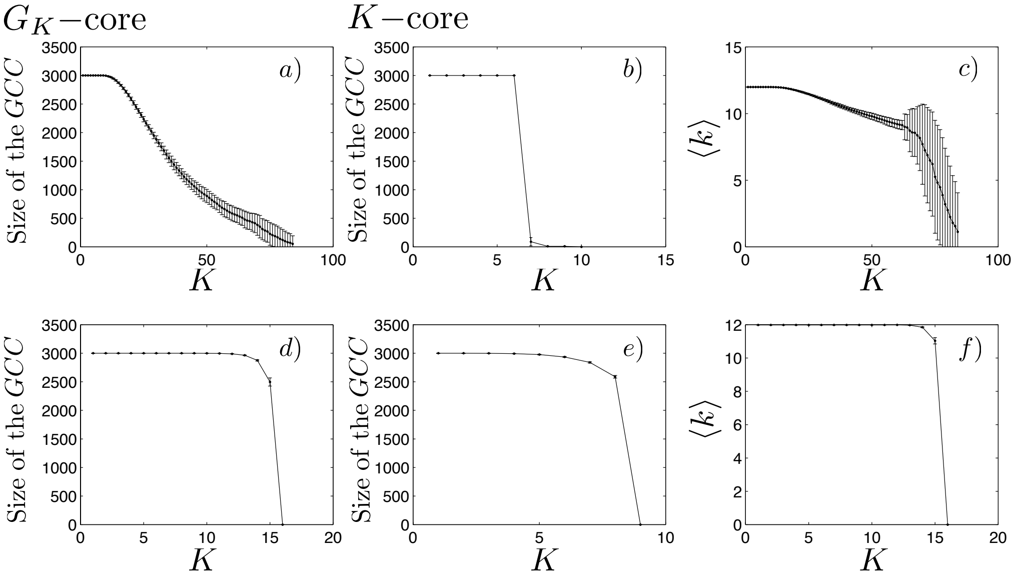

FIG. 1: Evolution of the size of the G_K-cores (a), K-cores (b) and the average connectivities of the G_K-cores as a function of the threshold K in a B-A ensemble containing 3000 nodes and with $\langle k \rangle = 12$. Evolution of the size of the G_K-cores (d), K-cores (e) and the average connectivities of the G_K-cores (f) as a function of the threshold K in a ER ensemble containing 3000 nodes and with $\langle k \rangle = 12$. See text for details.

- its degree is lower than K and
- at most 1 of its nearest neighbors has degree equal or higher than K.

If we iteratively apply this operation over a finite graph $\mathcal{G}$,

$$H_K^n(\mathcal{G}) = \overbrace{H_K \circ ... \circ H_K}^{n}(G),$$

we will reach a value, $n = N$, by which $(\forall M > N)$ $H_K^N(\mathcal{G}) = H_K^M(\mathcal{G})$. We can take it as a definition of the *generalized* K*-core*, by saying that:

$$G_K(\mathcal{G}) = H_K^N(\mathcal{G}). \qquad (4)$$

The equivalence between this definition and the one provided above can be easily checked: Indeed, on one hand, the algorithm itself forbids the presence of a node which is neither a hub or a connector, because, thank to its iterative nature, it only stops when all *surviving* nodes satisfy the conditions to belong to the G_K. On the other hand, we observe that the set isolated by the iterative algorithm is maximal: If a node (or a set of nodes) satisfies the conditions imposed by the algorithm, it is not pruned. We observe that, in any finite graph, $\exists K^*$ by which although $G_{K^*} \neq \varnothing$, $(\forall K > K^*)$ $G_K(\mathcal{G}) = \varnothing$. We will refer to $G_{K^*}(\mathcal{G})$ as the *deepest* G_K-core of $\mathcal{G}$.

Due to the potential richness of connectivity patterns that are allowed inside the G_K-core, we can categorize its nodes according to their topological roles:

- $G_K^{\mathrm{Con}}(\mathcal{G})$ is the set of nodes of $G_K(\mathcal{G})$ whose degree is lower than K, the K*-connectors*,
- $G_K^{\mathrm{Hub}}(\mathcal{G})$ is the set of nodes of $G_K(\mathcal{G})$ whose degree is equal or higher than K, the K*-hubs*.
- The set of K*-critical connectors*. A K-critical connector is a K-connector whose removal implies the breaking of the $G_K(\mathcal{G})$ in two or more parts. We can analogously define the set of K-critical hubs.

D. The K-core subgraph

For the K-core definition and the exploration of its interesting properties, we refer the interested reader to [3–5]. The K-core of a given graph $\mathcal{G}$, $KC(\mathcal{G})$, is the maximal induced subgraph whose nodes have degree *at least* K. It

can be obtained through the application of an algorithm qualitatively close to the one described above by iteratively removing nodes whose degree is lower than K. The sequence

$$... \subseteq KC(\mathcal{G}) \subseteq (K-1)K(\mathcal{G}) \subseteq ... \subseteq 2C(\mathcal{G}) \subseteq \mathcal{G}.$$

is the KC-decomposition sequence, and the largest K by which $KC(\mathcal{G}) \neq \varnothing$, the *deepest* K-core, will be referred to as $K^*C(\mathcal{G})$.

E. The M-core subgraph

We end this section by describing the M-core subgraph. We refer the interested reader to [7]. The M-core of a given graph $\mathcal{G}$, $M(\mathcal{G})$ is the maximal induced subgraph whose *links* participate, *at least* in M triangles. The M-core can be obtained by the application of an iterative algorithm as the one presented above which iteratively removes links participating in less than M triangles. Although it has not been used for the particular purposes of the current study, we can also define a M-core decomposition sequence in the same way we did with the G_K-core and the K-core.

II. MODEL NETWORKS

We now explore the behavior of the G_K decomposition of two standard models of random graphs, namely, the *Erdös-Rényi* (ER) graph [1] and the *Barabási-Albert* (BA) graph [6]. For every type of graph we create an ensemble of 100 networks each, with $\langle k \rangle = 12$ in both the BA and the ER ensemble. We compute the evolution of the *Giant Connected Component* of all the non-empty G_K-cores and K-cores of the corresponding decomposition sequences and we plot them as a function of the threshold defined by K, see Fig. 1. For the BA scale-free networks, we observe a long decomposition sequence, thereby obtaining a picture of the core topology of the net at many different levels, see Fig. 1a.The behavior of the G_K-decomposition sequence for the ER ensemble shows that the G_K-core is either the whole graph or empty which can be due to the almost uniform degree distribution of this kind of graph see Fig. 1d. The behavior of the two ensembles is qualitatively similar under the KC-decomposition, showing an all-to-nothing transition at values close to $\langle k \rangle/2$, see Fig. 1b,e. The average degree of the successive G_K-core subgraphs shows a slightly descending trend, whereas it remains constant in the case of the ER ensemble, mainly because, if the G_K-core is non-empty, it contains almost the whole graph. The counterintuitive decay in $\langle k \rangle$ for the BA ensemble can be explained by the increasing relative abundance of connectors against hubs within G_K as long as K increases.

III. INDICATORS OF PERFORMANCE

We explored the behavior of 7 quantitative indicators of social performance within the 'Pardus' game (www.pardus.at):

- *Experience* is a numerical indicator accounting for the experience of the player, related to battles in which the player has participated, or the number monsters he/she *killed*.
- *Activity* is a numerical indicator related to the number of actions performed by the player.
- *Age* is the number of days after the player joined the game,
- *Wealth*, numerical indicator accounting for the wealth of the player within the game. Wealth accounts for cash money, value of the equipment the player owns within the game.
- *Fraction of leaders* fraction of players who are leaders in some aspect in a given alliance. Alliance should not be confused with nations. Alliances are small, organized groups of players. In the studied universe, we identify around ~ 140 different alliances. Every alliance has its own *local* leaders.
- *Global leadership* is numerical indicator evaluating the degree of leadership of the player. It is increased by doing missions, which are mainly transporting goods or killing monsters. The higher the *Global leadership*, the more powerful items may be bought – and the more missions are required to reach the next level. In general we can say that the higher this indicator, the more powerful and influential is the player within the whole society defined by the game.
- *Gender composition* evaluates the fraction of males within a given group of players.

In the table we show the scores of all these indicators for all 7 studied graphs at their respective $\tilde{K}$ and K^*-levels. We distinguish between connectors and hubs. We compute theses social indicators for the K^*-core as well.c The last column *NumberInd* is the number of individuals of the observed subset of nodes.

In tables I and II we provide the above mentioned social indicators of the nodes belonging to i) the critical G_K-cores and their hubs, ii) the deepest G_K-cores and their hubs, iii) the deepest K-core subgraphs and the whole networks.

IV. SOCIAL NETWORKS OF THE 'PARDUS' VIRTUAL SOCIETY

Throughout the paper we based our analysis in three social networks, namely:

- Communication network: A link between two players is established if they had a communicative interaction (a player sent a message to the other player, regardless the direction of the informative exchange) within the period under study.
- Trade network: A link is established if two players had a commercial relation within the period under study.
- Friendship network: A link is established if a given player identifies the other as 'friend'. This identification an be previous to the period of study; each player has a list of those players who are tagged as friends within the virtual society.

We studied two periods of time: i) from day 796 to day 856 and ii) from day 1140 to day 1200; being the day '0' the day in which the game was launched. In figures 2-8 we study the evolution of basic statistical indicators of the studied networks and the intersections we can extract from them. In black we have the behaviour of the real networks, in read, the average behaviour of an ensemble of 25 randomised versions of the original one. Networks obtained through intersection are randomised *after* performing the intersection. Otherwise, it is likely that we would end up quickly to empty networks.

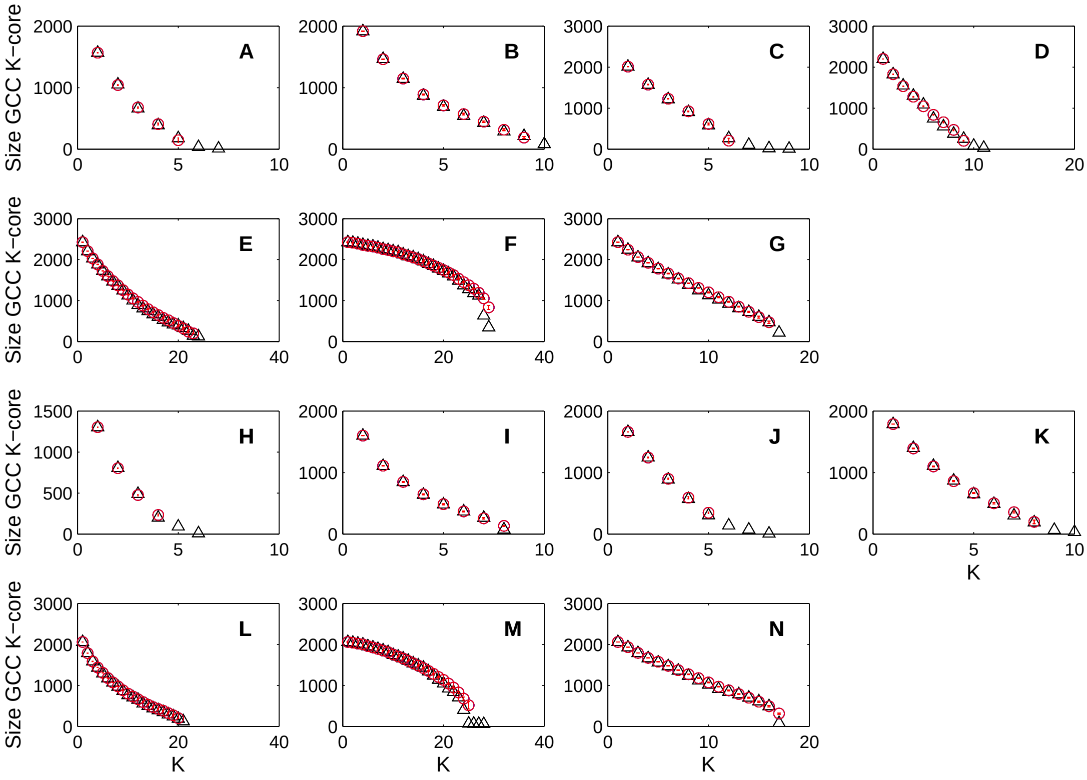

FIG. 2: Evolution of the size of the Giant Connected Component of the K-core for the networks corresponding to the period 796-856 as a function of K. a) $\mathcal{G}_{FTC}$, b) $\mathcal{G}_{FC}$, c) $\mathcal{G}_{FT}$, d) $\mathcal{G}_{TC}$, e) $\mathcal{G}_{C}$, f) $\mathcal{G}_{T}$, g) $\mathcal{G}_{F}$ and the networks corresponding to the period 1140–1200, h) $\mathcal{G}_{FTC}$, i) $\mathcal{G}_{FC}$, j) $\mathcal{G}_{FT}$, k) $\mathcal{G}_{TC}$, l) $\mathcal{G}_{C}$, m) $\mathcal{G}_{T}$, n) $\mathcal{G}_{F}$. Black triangles depict the behaviour of real networks, red circles and their associated error bars depict the average behaviour of an ensemble of 25 randomised versions of the original networks.

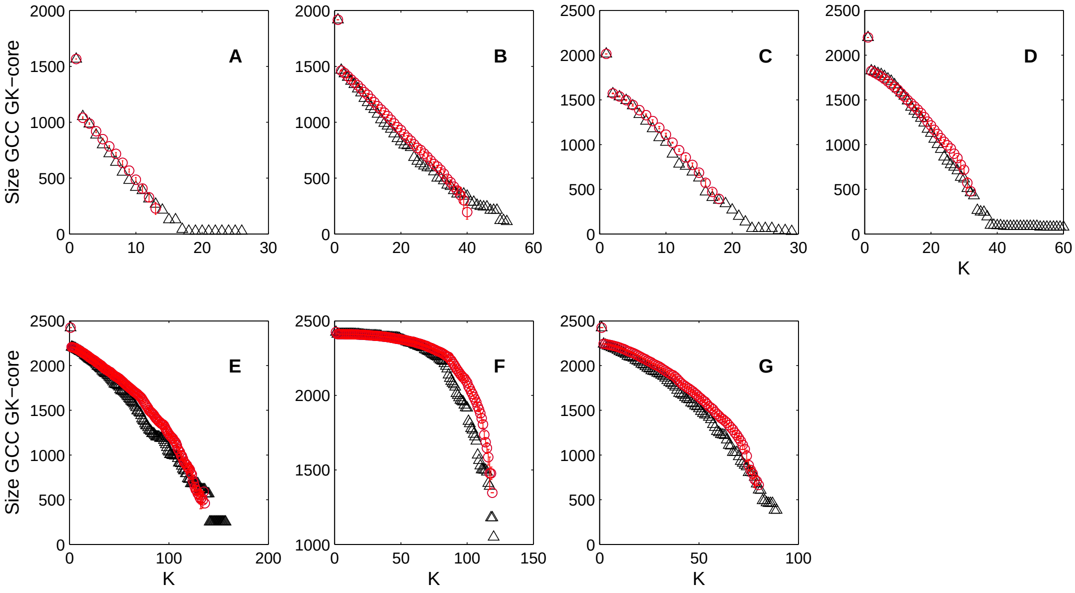

FIG. 3: Evolution of the size of the Giant Connected Component of the GK-core for the networks corresponding to the period 796–856 as a function of K. a) $\mathcal{G}_{FTC}$, b) $\mathcal{G}_{FC}$, c) $\mathcal{G}_{FT}$, d) $\mathcal{G}_{TC}$, e) $\mathcal{G}_{C}$, f) $\mathcal{G}_{T}$, g) $\mathcal{G}_{F}$. Black triangles depict the behaviour of real networks, red circles and their associated error bars depict the average behaviour of an ensemble of 25 randomised versions of the original networks.

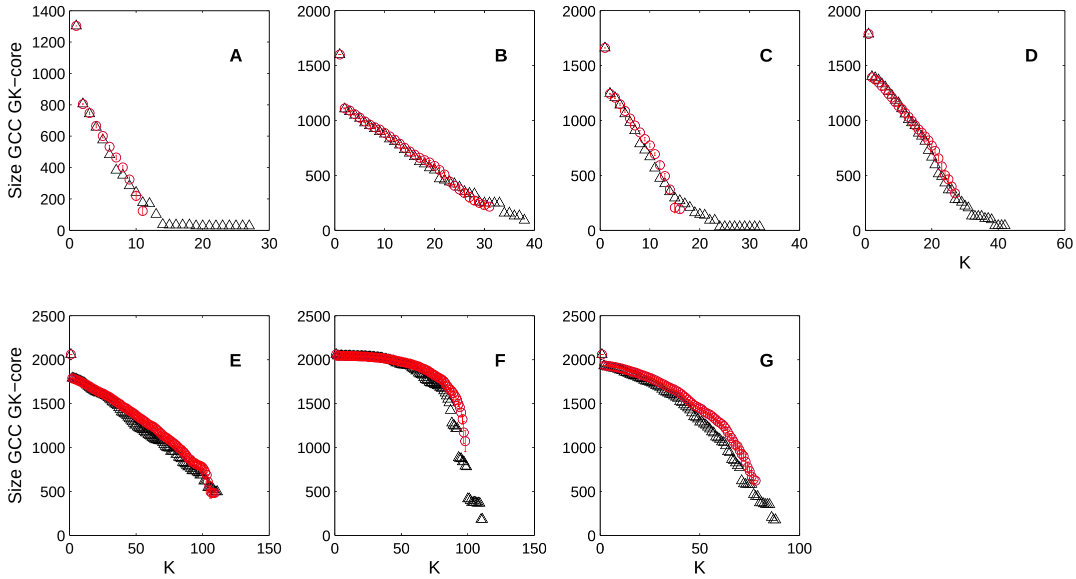

FIG. 4: Evolution of the size of the Giant Connected Component of the GK-core for the networks corresponding to the period 1140–1200 as a function of K. a) $\mathcal{G}_{FTC}$, b) $\mathcal{G}_{FC}$, c) $\mathcal{G}_{FT}$, d) $\mathcal{G}_{TC}$, e) $\mathcal{G}_{C}$, f) $\mathcal{G}_{T}$, g) $\mathcal{G}_{F}$. Black triangles depict the behaviour of real networks, red circles and their associated error bars depict the average behaviour of an ensemble of 25 randomised versions of the original networks.

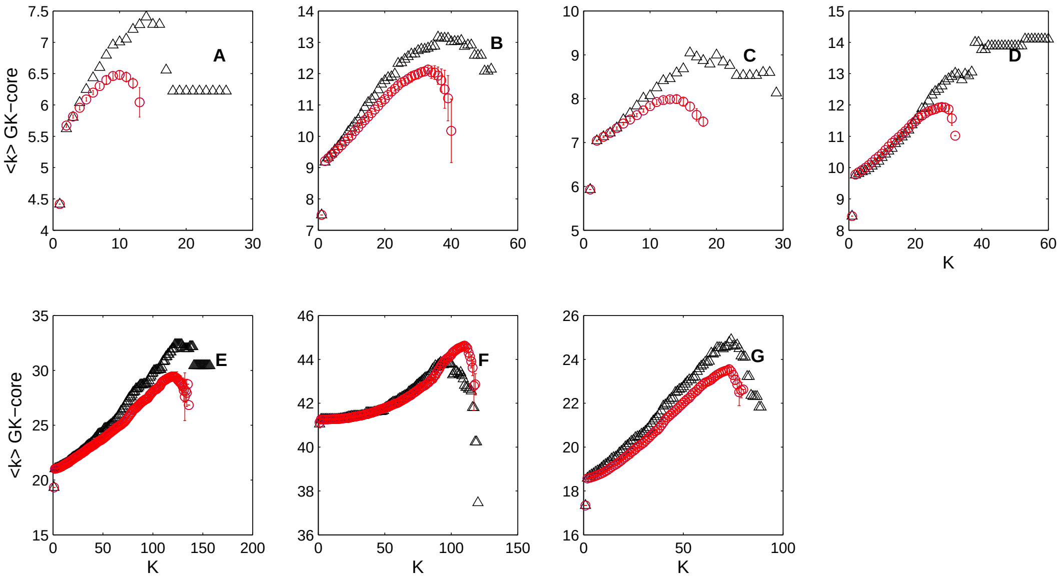

FIG. 5: Evolution of the average degree of the Giant Connected Component of the GK-core for the networks corresponding to the period 796–856 as a function of K. a) $\mathcal{G}_{FTC}$, b) $\mathcal{G}_{FC}$, c) $\mathcal{G}_{FT}$, d) $\mathcal{G}_{TC}$, e) $\mathcal{G}_{C}$, f) $\mathcal{G}_{T}$, g) $\mathcal{G}_{F}$. Black triangles depict the behaviour of real networks, red circles and their associated error bars depict the average behaviour of an ensemble of 25 randomised versions of the original networks.

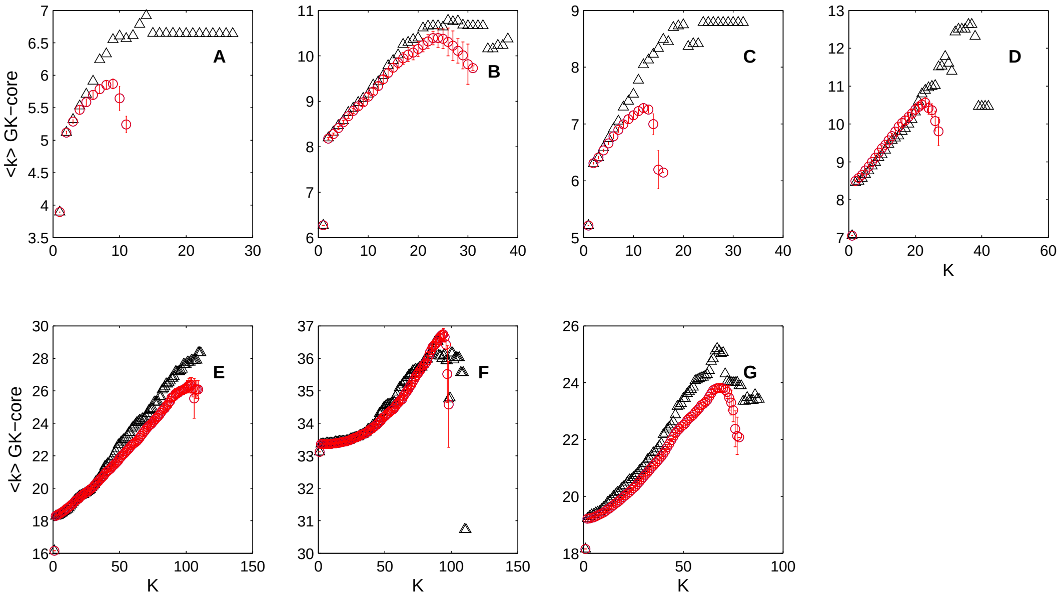

FIG. 6: Evolution of the average degree of the Giant Connected Component of the GK-core for the networks corresponding to the period 1140–1200 as a function of K. a) $\mathcal{G}_{FTC}$, b) $\mathcal{G}_{FC}$, c) $\mathcal{G}_{FT}$, d) $\mathcal{G}_{TC}$, e) $\mathcal{G}_{C}$, f) $\mathcal{G}_{T}$, g) $\mathcal{G}_{F}$. Black triangles depict the behaviour of real networks, red circles and their associated error bars depict the average behaviour of an ensemble of 25 randomised versions of the original networks.

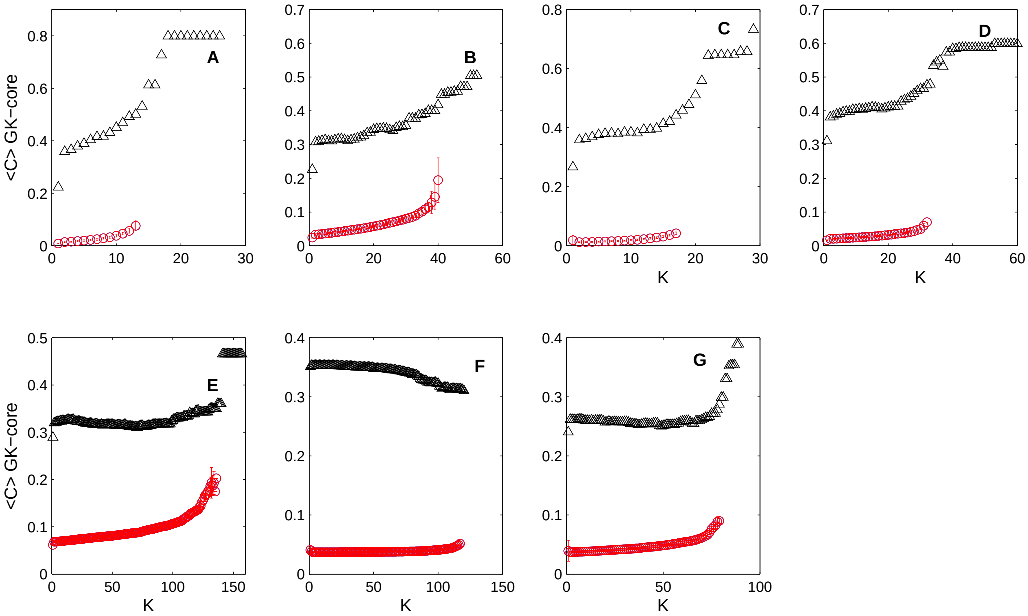

FIG. 7: Evolution of the average clustering coefficient of the Giant Connected Component of the GK-core for the networks corresponding to the period 796–856 as a function of K. a) $\mathcal{G}_{FTC}$, b) $\mathcal{G}_{FC}$, c) $\mathcal{G}_{FT}$, d) $\mathcal{G}_{TC}$, e) $\mathcal{G}_{C}$, f) $\mathcal{G}_{T}$, g) $\mathcal{G}_{F}$. Black triangles depict the behaviour of real networks, red circles and their associated error bars depict the average behaviour of an ensemble of 25 randomised versions of the original networks.

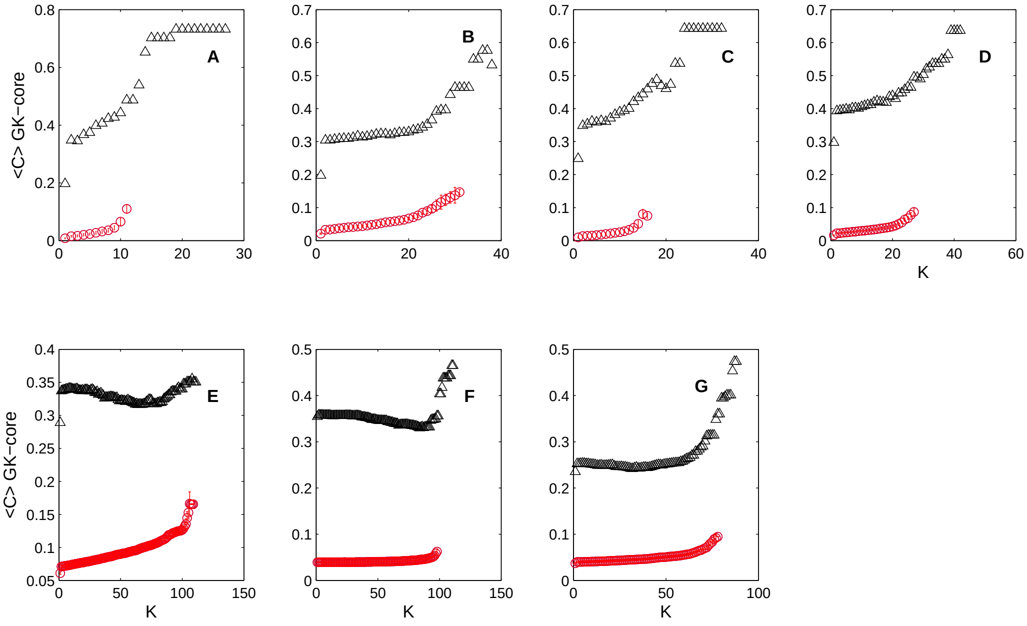

FIG. 8: Evolution of the average degree of the Giant Connected Component of the GK-core for the networks corresponding to the period 1140–1200 as a function of K. a) $\mathcal{G}_{FTC}$, b) $\mathcal{G}_{FC}$, c) $\mathcal{G}_{FT}$, d) $\mathcal{G}_{TC}$, e) $\mathcal{G}_{C}$, f) $\mathcal{G}_{T}$, g) $\mathcal{G}_{F}$. Black triangles depict the behaviour of real networks, red circles and their associated error bars depict the average behaviour of an ensemble of 25 randomised versions of the original networks.

TABLE I: Table with the social indicators. Period 796-856

	⟨Experience⟩	⟨Activity⟩	⟨Age⟩	⟨Wealth⟩	gendComp	FracLead	⟨GlobalLead⟩	NInd
$\mathcal{G}_{FCT}$								
Characteristic GK	4.9×10^5	3.6×10^6	684	5.37×10^7	0.809	0.127	8.53	110
Hubs	6.03×10^5	4.57×10^6	735	5.89×10^7	0.947	0.526	9.32	19
Deepest GK	6.24×10^5	4.45×10^6	777	7.89×10^7	0.88	0.08	10	25
Hubs	1.56×10^6	6.14×10^6	855	8.03×10^7	1	1	13	2
Deepest K-Core 1	4.34×10^5	3.7×10^6	675	4.88×10^7	0.7	0.3	9.1	10
All Net	3.59×10^5	2.81×10^6	615	3.19×10^7	0.871	0.165	6.84	1564
$\mathcal{G}_{FC}$								
Critical GK	3.77×10^5	2.97×10^6	639	3.45×10^7	0.875	0.14	6.96	784
Hubs	5.51×10^5	3.8×10^6	690	4×10^7	0.885	0.33	8.24	288
Deepest GK	5.71×10^5	3.71×10^6	691	4.64×10^7	0.907	0.215	8.78	107
Hubs	8.68×10^5	4.97×10^6	718	4.26×10^7	1	0.8	9.4	5
Deepest K-Core 2	7.11×10^5	4.36×10^6	724	5.16×10^7	0.841	0.305	9.18	82
All Net	3.43×10^5	2.7×10^6	610	2.91×10^7	0.868	0.145	6.63	1915
$\mathcal{G}_{FT}$								
Characteristic GK	4.6×10^5	3.39×10^6	688	4.62×10^7	0.797	0.138	8.08	123
Hubs	6.54×10^5	4.8×10^6	792	6.02×10^7	0.929	0.429	8.86	14
Deepest GK	6.14×10^5	4.41×10^6	786	7.42×10^7	0.857	0.107	10.2	28
Hubs	1.56×10^6	6.14×10^6	855	8.03×10^7	1	1	13	2
Deepest K-Core 3	4.23×10^5	3.71×10^6	687	5.53×10^7	0.727	0.273	8.91	11
All Net	3.37×10^5	2.68×10^6	618	2.89×10^7	0.871	0.137	6.64	2012
$\mathcal{G}_{CT}$								
Characteristic GK	3.63×10^5	2.8×10^6	615	3.02×10^7	0.862	0.116	7.06	950
Hubs	4.89×10^5	3.61×10^6	647	5.01×10^7	0.905	0.293	8.88	222
Deepest GK	6.04×10^5	4.04×10^6	745	5.93×10^7	0.947	0.158	10.1	76
Hubs	1.09×10^6	5.36×10^6	835	9.25×10^7	1	0.333	11.3	3
Deepest K-Core 4	6.3×10^5	4.2×10^6	741	7.85×10^7	0.971	0.176	10	34
All Net	3.18×10^5	2.57×10^6	606	2.72×10^7	0.872	0.128	6.55	2196
$\mathcal{G}_{C}$								
Deepest GK	5.37×10^5	3.68×10^6	693	4.22×10^7	0.915	0.23	8.15	248
Hubs	9.71×10^5	5.14×10^6	709	5.18×10^7	1	0.5	8.5	4
Deepest K-Core	6.67×10^5	4.24×10^6	716	4.63×10^7	0.93	0.279	8.76	129
$\mathcal{G}_{T}$								
Deepest GK	4.27×10^5	3.11×10^6	657	3.45×10^7	0.884	0.149	7	1019
Hubs	5.17×10^5	4.15×10^6	766	6.69×10^7	0.733	0.167	9.1	30
Deepest K-Core	3.82×10^5	2.88×10^6	600	3.34×10^7	0.896	0.127	8.49	347
$\mathcal{G}_{F}$								
Characteristic GK	1.72×10^5	1.66×10^6	512	1.52×10^7	0.874	0.0402	5.69	697
Hubs	4.07×10^5	3.13×10^6	687	3.35×10^7	0.87	0.171	6.96	1487
Deepest GK	5.34×10^5	3.6×10^6	712	4.3×10^7	0.868	0.196	7.31	372
Hubs	9.32×10^5	4.96×10^6	807	6.18×10^7	0.818	0.455	10.1	11
Deepest K-Core	6.81×10^5	4.33×10^6	775	4.85×10^7	0.881	0.252	8.1	218
All Players	3.07×10^5	2.5×10^6	606	2.59×10^7	0.87	0.119	6.39	2422

TABLE II: Table with the social indicators. Period 1140-1200

	⟨Experience⟩	⟨Activity⟩	⟨Age⟩	⟨Wealth⟩	*gendComp*	FracLead	⟨GlobalLead⟩	NInd
$\mathcal{G}_{FCT}$								
Characteristic GK	7.72×10^5	5.69×10^6	1.02×10^3	9.84×10^7	0.885	0.195	10.7	87
Hubs	1.01×10^6	6.86×10^6	1.08×10^3	1.23×10^8	0.933	0.4	11.4	15
Deepest GK	9.78×10^5	5.96×10^6	1.09×10^3	1.14×10^8	0.962	0.154	11.3	26
Hubs	5.69×10^5	7.39×10^6	1.2×10^3	3.03×10^8	1	1	12	2
Deepest K-Core	7.18×10^5	6.23×10^6	1.09×10^3	1.4×10^8	0.889	0.111	11	9
All Net	4.86×10^5	3.88×10^6	857	4.87×10^7	0.875	0.165	7.64	1303
$\mathcal{G}_{FC}$								
Characteristic GK	8.47×10^5	5.72×10^6	1.04×10^3	7.69×10^7	0.884	0.207	9.41	121
HUBS	1.32×10^6	6.96×10^6	1.15×10^3	1.24×10^8	0.778	0.333	12.6	9
Deepest GK	8.07×10^5	5.59×10^6	1.01×10^3	6.37×10^7	0.882	0.235	8.69	85
Hubs	1.53×10^6	6.84×10^6	1.13×10^3	7.26×10^7	0.714	0.143	12.7	7
Deepest K-Core	9.4×10^5	6.03×10^6	1.01×10^3	6.66×10^7	0.882	0.329	9.5	76
All Net	4.69×10^5	3.72×10^6	842	4.35×10^7	0.871	0.154	7.4	1600
$\mathcal{G}_{FT}$								
Characteristic GK	8.48×10^5	5.77×10^6	1.05×10^3	8.94×10^7	0.892	0.169	10.6	83
Hubs	1.34×10^6	7.37×10^6	1.13×10^3	1.8×10^8	0.889	0.333	12.1	9
Deepest GK	9.2×10^5	5.87×10^6	1.11×10^3	1.1×10^8	0.935	0.194	11.3	31
Hubs	5.69×10^5	7.39×10^6	1.2×10^3	3.03×10^8	1	1	12	2
Deepest K-Core	7.18×10^5	6.23×10^6	1.09×10^3	1.4×10^8	0.889	0.111	11	9
All Net	4.76×10^5	3.77×10^6	869	4.46×10^7	0.872	0.143	7.56	1660
$\mathcal{G}_{CT}$								
Characteristic GK	7.38×10^5	5.34×10^6	989	9.17×10^7	0.934	0.231	10.9	91
Hubs	4.68×10^5	6.88×10^6	1.11×10^3	1.7×10^8	1	0.6	11.8	5
Deepest GK	7.06×10^5	5.43×10^6	1.02×10^3	1×10^8	0.927	0.341	10.7	41
Hubs	2.98×10^5	5.87×10^6	982	4.03×10^7	1	0.5	11	2
Deepest K-Core	9.53×10^5	5.99×10^6	1.03×10^3	1.1×10^8	0.912	0.206	11.3	34
All Net	4.33×10^5	3.54×10^6	831	4.22×10^7	0.871	0.137	7.44	1788
$\mathcal{G}_{C}$								
Deepest GK	6.25×10^5	4.49×10^6	884	5.53×10^7	0.888	0.24	8.21	483
Hubs 5	1.21×10^6	6.74×10^6	1.03×10^3	6.25×10^7	0.929	0.429	10.1	14
Deepest K-Core	8.23×10^5	5.57×10^6	968	7.53×10^7	0.874	0.394	8.77	127
$\mathcal{G}_{T}$								
Deepest GK	4.27×10^5	3.11×10^6	657	3.45×10^7	0.884	0.149	7	1019
Hubs	5.17×10^5	4.15×10^6	766	6.69×10^7	0.733	0.167	9.1	30
Deepest K-Core	3.82×10^5	2.88×10^6	600	3.34×10^7	0.896	0.127	8.49	347
$\mathcal{G}_{F}$								
Characteristic GK	1.9×10^5	1.88×10^6	608	1.86×10^7	0.86	0.0457	6.08	328
Hubs	5.05×10^5	4×10^6	925	4.65×10^7	0.87	0.155	7.61	1585
Deepest GK	7.57×10^5	5.34×10^6	1.05×10^3	5.96×10^7	0.877	0.175	7.33	171
Hubs	1.39×10^6	6.68×10^6	1.15×10^3	7.8×10^7	0.6	0	12.8	5
Deepest K-Core	1×10^6	6.12×10^6	1.08×10^3	6.83×10^7	0.88	0.253	9.11	83
All players	4.3×10^5	3.5×10^6	841	3.96×10^7	0.87	0.12	7.51	2059

[1] M. E. Newman, S. H. Strogatz, and D. J. Watts, Phys Rev E **64** (2001).
[2] M. Szell and S. Thurner, Social Networks **39**, 313 (2010).
[3] S. B. Seidman, Social Networks **5**, 269 (1983).
[4] B. Bollobás, Graph Theory and Combinatorics, Proc Cambridge Combinatorial Conf in honor to Paul Erdös, Academic press pp. 35–57 (1984).
[5] S. N. Dorogovtsev, A. V. Goltsev, and J. F. F. Mendes, Phys Rev Lett **96**, 040601 (2006).
[6] A.-L. Barabási and R. Albert, Science **286**, 509 (1999).
[7] Colomer-de-Simón, P, Serrano, M Á, Beiró, M G, Alvarez-Hamelin, J I, Boguñá Deciphering the global organization of clustering in real complex networks. Nat Sci Rep **3**:2517 doi:10.1038/srep02517 (2013)
